# Recurrent rectal cancer cured by transsacral prone longitudinal incision combined with bilateral gluteus maximus “kite” flap filling: a case report

**DOI:** 10.3389/fonc.2025.1550960

**Published:** 2025-08-27

**Authors:** Lixia Zhang, Xiaoling Jiang, Maoyuan Wu, Wenwen Zhang, Guanyan Wang, Wei Yang, Tingchao Li, Lianli He, Gangcheng Wang

**Affiliations:** ^1^ Department of Gynecology and Obstetrics, The Third Affiliated Hospital of Zunyi Medical University (The First People’s Hospital of Zunyi), Zunyi, Guizhou, China; ^2^ Department of Imaging, The Third Affiliated Hospital of Zunyi Medical University (The First People’s Hospital of Zunyi), Zunyi, China; ^3^ Department of Pathology, The Third Affiliated Hospital of Zunyi Medical University (The First People’s Hospital of Zunyi), Zunyi, China; ^4^ Department of Abdominal and Pelvic Tumor Surgery, The First Affiliated Hospital of Zhengzhou University, Zhengzhou, China

**Keywords:** local recurrence of rectal cancer, radical resection, longitudinal incisional, kite flap, the posterior wall of the vagina

## Abstract

Locally recurrent rectal cancer (LRRC) refers to the lesions that appear in the pelvic cavity and perineum with the same pathological type as the primary tumor after radical operation of rectal cancer, excluding other distant metastases such as liver, lung, and bone. Radical surgical resection in such patients is a central element in improving quality of life andsurvival. In this paper, we report the case of a patient who was admitted to our hospital with a recurrence of ulcerated moderately differentiated carcinoma of the lower rectum with vaginal involvement after comprehensive treatment. After discussion by a multidisciplinary team, a transsacral prone longitudinal incision combined with bilateral gluteus maximus “kite” flap padding was used to remove the tumor tissue completely, and the postoperative area healed well, which improved the patient’s quality of life and increased her survival rate.

## Background

The common parts of local recurrence after comprehensive treatment for rectal cancer are mainly anastomosis, perineum, soft tissues in the pelvis and adjacent organs or structures. For different recurrence sites, treatment programs and surgical methods are different.

Clinically, surgical operation is difficult due to the complexity of the pelvic anatomy and the disruption of normal anatomical structures by the first surgery or radiotherapy. Here, we report the diagnosis and management of a case of ulcerated moderately differentiated adenocarcinoma of the lower rectum with recurrence and vaginal involvement after comprehensive treatments.

## Case presentation

A 45-year-old female patient with “irregular vaginal discharge for 1+ months” was admitted to the hospital. One month ago, she presented with irregular vaginal discharge, which was yellowish, watery and occasionally bloody. Family history: Patients with a history of similar illnesses and a family genetic predisposition to diseases similar to the patient were excluded.

Her history was as follows: 1+ years ago (April 2022), because of alternate changes in bowel symptoms, manifested as diarrhea and constipation alternately, an average of 3-4 times/day, accompanied by the thinning of stools. After ruling out surgical contraindications at the other hospital, a “laparoscopic perineal resection was combined with radical rectal cancer resection and sigmoidostomy” was performed on 22nd July 2022. During the operation, the tumor was located in the lateral posterior wall of the low rectum, approximately 5.5*4.6*1.3 cm in size, approximately 1.5 cm from the anal verge, and infiltrated to the dentate line, occupying 2/3 of the intestinal lumen. The tumor seemed to invade through the whole intestinal wall, and there was no obvious abnormality of the peritoneum of the pelvic floor. Multiple enlarged lymph nodes were observed around the inferior mesenteric vessels. Postoperative pathology revealed the following: rectal tumor resection specimen; ulcerated moderately differentiated adenocarcinoma with large necrosis; cancer invading the serosal layer; and cancerous embolus and nerve invasion in the vasculature. No cancerous tissue was seen at the proximal or distal margins, and metastasis of cancerous tissue was detected in the peri-intestinal lymph nodes(2/18). Pathological AJCC stage: pT4N1b. Six courses of chemotherapy (oxaliplatin + capecitabine) were administered between September 2022 and April 2023 after surgery, during which radiotherapy was administered after the second course of chemotherapy. The AFP, CEA, and CA19–9 levels were detected within the normal reference range during radiotherapy; pelvic MRI in February 2023 suggested a circular enhancing nodular shadow of approximately 1.0 cm in transverse diameter in the posterior aspect of the vagina, with no obvious diffusion restriction. In August 2023, she returned to the hospital for follow-up, and the tumor indicators were within the normal reference range. Pelvic MRI suggested that a ring-shaped nodular shadow with a transverse diameter of approximately 1.5 cm was seen present in the posterior part of the vagina, and diffusion was limited.

**Figure 1:**
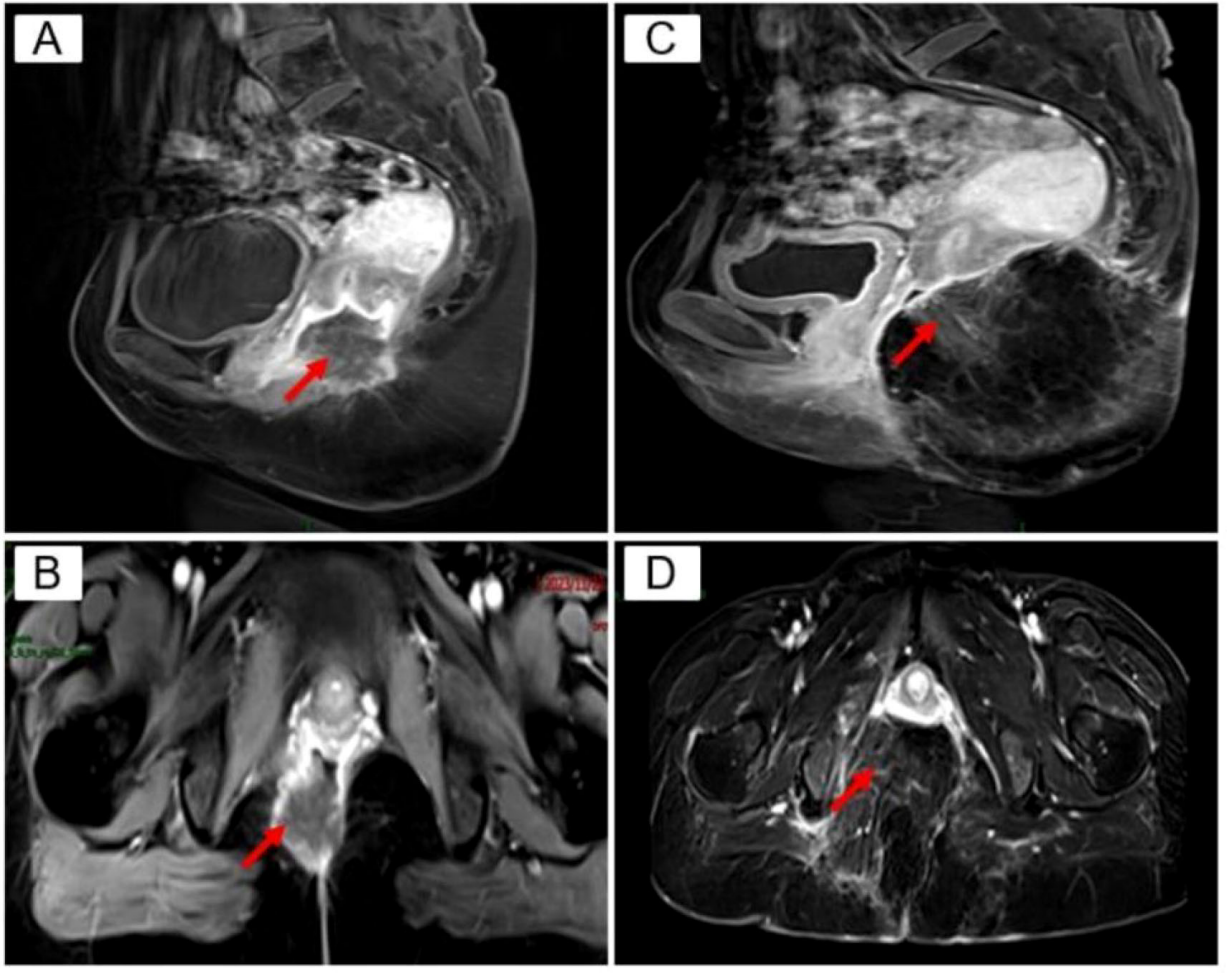
Pelvic MRI. red arrows **(A)** Preoperative TIWI Cor: annular enhancing mass in the posterior vaginal wall, transverse diameter of about 3.2 3.8 cm, irregular borders, lobulated, seems to see burrs; **(B)** Preoperative T1 WI Sag: transverse diameter of about 4.0 cm; **(C)** Postoperative T1W1 Cor: caudal bone is absent, and the mass is normally altered after resection; **(D)** Postoperative TI WI Sag.

Auxiliary examination: Positron emission tomography-computed tomography (PET/CT) revealed a mass with a soft tissue density shadow in the anal region, an unclear border, and unclear boundaries with the adjacent vagina. FDG-PET revealed increased radioactivity uptake, with an SUVmax of 14.1 and an SUVavg of 6.87, and the range of the largest region was approximately 3.6*3.4 cm. Involvement of the vagina was possible, and the remaining tissues and organs did not exhibit clear abnormal hypermetabolic foci. Pelvic Magnetic resonance imaging (MRI) + enhanced MRI revealed a ring-shaped intensified mass in the posterior vaginal wall, and the metastatic tumor was likely to be large. The posterior wall of the vagina had a transverse diameter of approximately 3.2*3.8 cm, the sagittal position of the circular ring-shaped intensified mass shadow was approximately 4.0 cm, the border was irregular and lobulated, and the burr was clearly visible (**Figures 1A, B**).

Compared with the MRI image obtained on August 08, 2023, the nodule in the posterior wall of the vagina was obviously enlarged. Biopsy pathology of the posterior vaginal wall suggested (tissue of the posterior vaginal wall) a large amount of coagulative necrosis and a small amount of adenocarcinoma tissue. Laboratory tests revealed a squamous epithelial cell carcinoma antigen concentration of 0.8 ng/ml.

**Figure 2:**
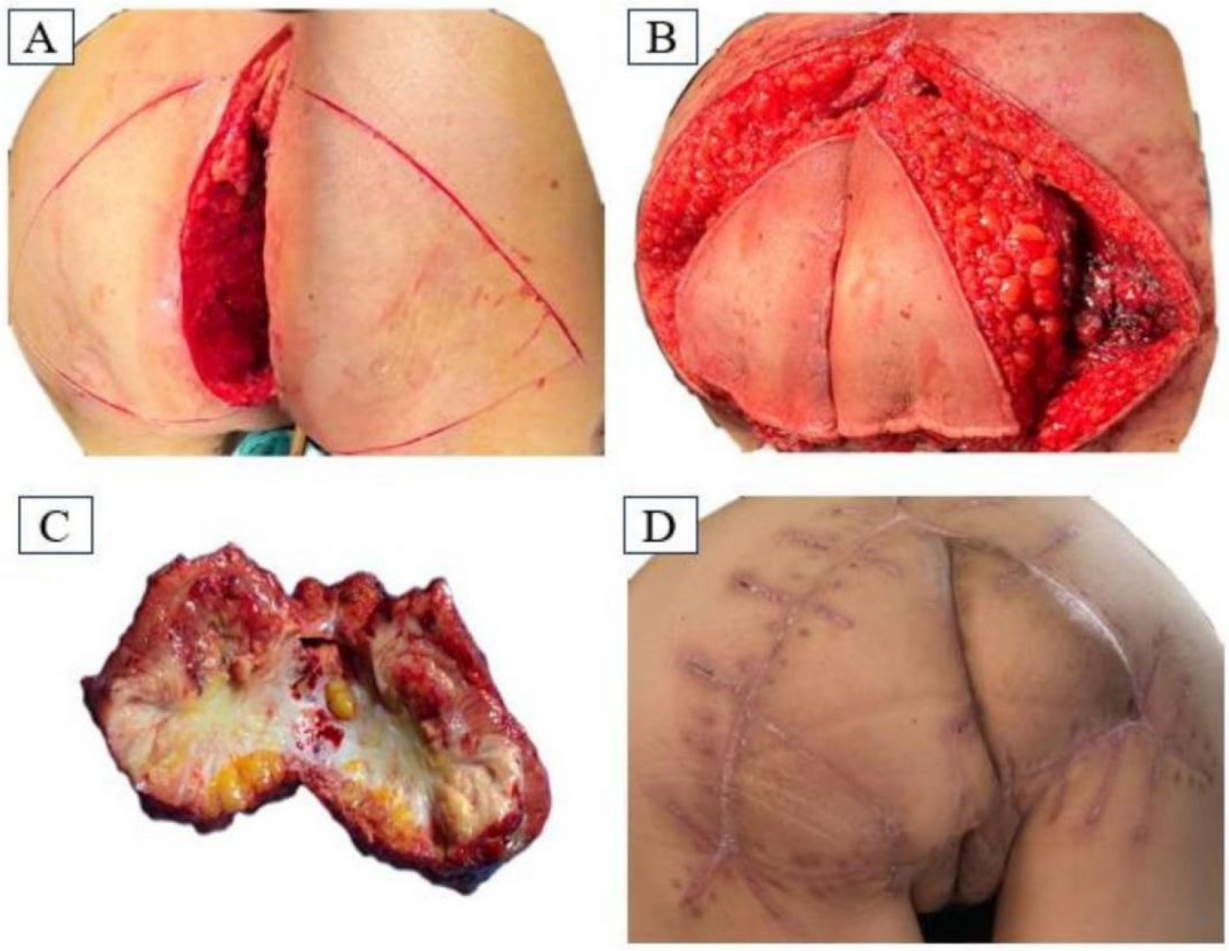
**(A)** Longitudinal incision approach in prone position; **(B)** Intraoperative flap design; **(C)** Surgical removal of the mass specimen; **(D)** Recovery of the operated area at 38 days postoperatively.

After discussion by our multidisciplinary team, comprehensive assessment of the patient’s local recurrence of tumor tissue could be performed via radical resection. On December 9, 2023, under anesthesia, tumor resection of the posterior vaginal wall + caudal osteotomy + bilateral gluteus maximus kite flap excision and grafting was carried out, and the patient was placed in a prone position. When anesthesia was in effect, the towel was routinely disinfected and spread out, a longitudinal incision was made along the gluteal groove, up to the third sacrum, and down to the external portion of the vagina, and the subcutaneous tissue behind the coccyx was incised through the sacrum surface. The left hand entered the vagina, and the tumor was found to be located in the posterior wall of the vagina adhering to sacral vertebra 5 and the coccyx. The gluteus maximus muscle was dissected from the sacrococcygeal attachment point below sacral vertebra three, and sacral vertebra 5 was dissected. The anus tibialis muscle and the two sides of the vagina and the posterior wall were dissected under the guidance of the hand in the vagina at a distance of 3.0 cm from the tumor to retain the anterior wall of the vagina under the urethra, and the upper boundary reached the posterior fornix of the vagina. The tissues ofthe left and right sides were pulled toward the central defect of the pelvic floor, and the incision could not be sutured with high tension, so a tipped flap graft was generated. The gluteal midline was taken as the side length, and a symmetrical “triangle” mark was made on the surface of the gluteus maximus muscle on both sides, in which the skin and subcutis of the other two sides were incised to reach the gluteus maximus muscle. The superior gluteal artery was used as the tip of the flap, part of the gluteus maximus muscle was freed, the flap was cross-folded and filled into the incision, the excess skin was resected, the skin was closed, and a drainage tube was placed under the left and right sides of the vagina at the flaps. A drainage tube was placed under the right and left vaginal flaps (**Figure 2**).

**Figure 3:**
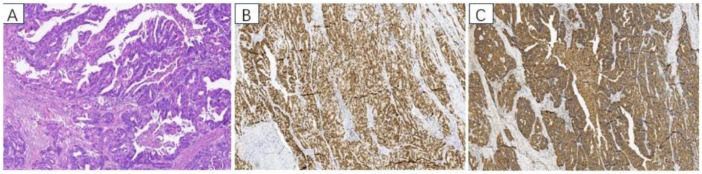
Pathological findings: **(A)** HE staining of adenocarcinoma, adenocarcinoma cells formed irregular adenoid structures, and pathologic nuclear schizophrenia was common. Immunohistochemistry: **(B)** CDX2 positive staining, **(C)** CK20 positive staining.

Postoperative pathology revealed adenocarcinoma. The immunohistochemistry results were as follows: CK8/18 (+), CK20 (+), CDX-2 (+), Villin (+), p16 (+), EGFR (+), P53 (-), CK7 (-), CD56 (-), CA125 (-), WT1 (-), ER (-), PR (-), CgA (foci +), Syn (-), and Ki-67 (+) (approximately 70%) (**Figure 3**).

**Figure 4:**
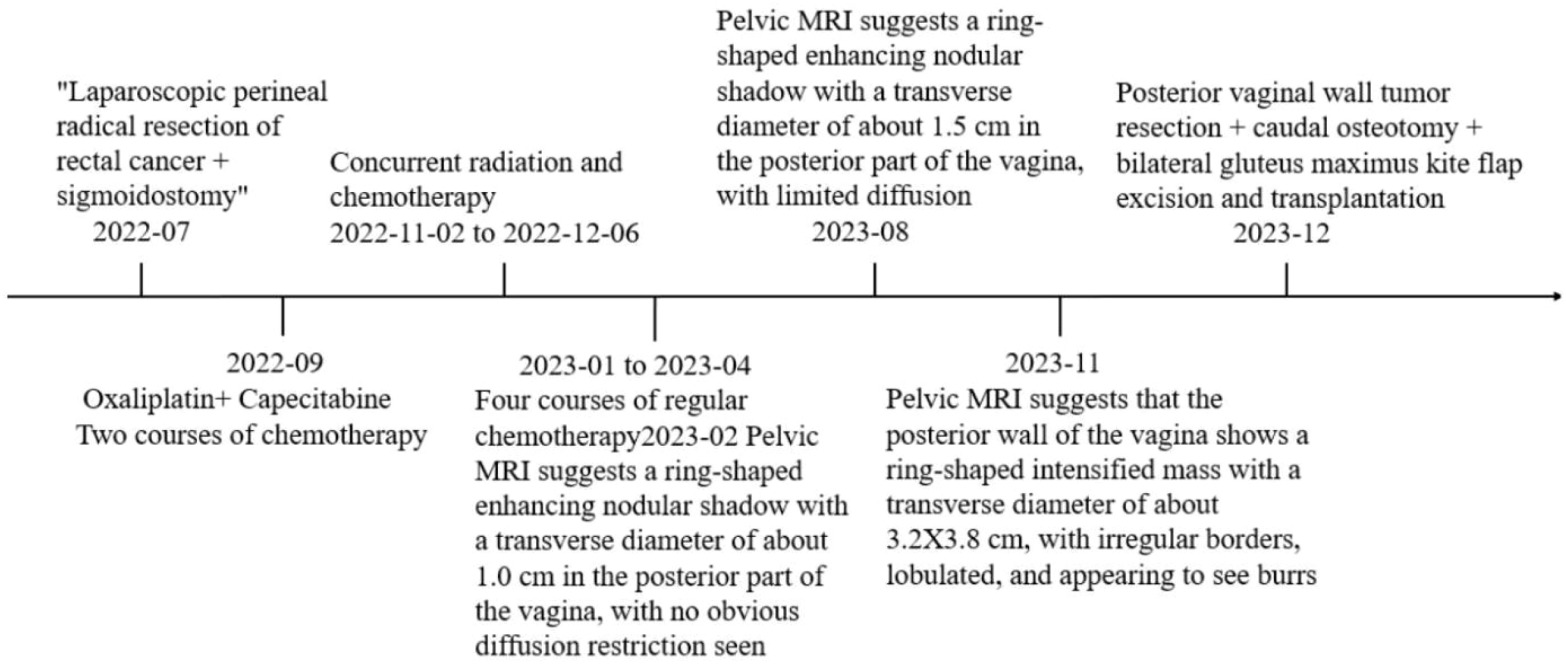
Timeline of the patient.

She returned to the outpatient clinic for review 38 days after the operation, and her examination showed that the operation area of the buttocks was well recovered. Pelvic MRI scanning + enhancement suggested postoperative changes after resection of the mass in the posterior vaginal wall, and part of the tailbone was missing; the rest ofthe patient did not show any abnormalities (**Figures 1C, D**). The timeline of the patient's treatment process was depicted in **Figure 4**.

## Discussion and conclusions

With the extensive multidisciplinary and comprehensive treatment of rectal cancer, thelocal recurrence rate of rectal cancer has decreased to less than 10% (1, 2). Locally recurrent recta cancer (LRRC) refers to foci that appear in the pelvis and perineum region with the same pathologic type as the primary tumor after radical surgery for rectal cancer, excluding the accompanying other distant transitions, such as liver, lungs and bones. The common sites of local recurrence are mainly the anastomosis, perineum, soft tissues in the pelvis and adjacent organs or structures. Due to the heterogeneity of the patient population, different clinical manifestations, and different sites of recurrent tumor tissue invasion, there is a lack of standardized diagnostic and therapeutic procedures for the diagnosis, treatment strategies, and surgical methods of LRRC. In recent years, studies at home and abroad have shown that the treatment of local recurrence in patients with rectal cancer should be individualized and based on a multidisciplinary team to carry out diagnosis and treatment (3), and radical surgical resection for local recurrence (R0 resection) is still the best choice for successful treatment of local recurrence (4).

Close follow-up after radical rectal cancer treatment is an important means to diagnose recurrence as early as possible, and regular tumor marker tests, imaging examinations and physical examinations are more important for patients with recurrence and no symptoms. Preoperative radiographic evaluation of pelvic wall involvement is a key factor in surgical decision-making, ruling out distant metastases, determining the likelihood of tumor R0 resection, and helping to plan and extent resection (5, 6). Besides, Histopathological findings confirm that homology with previous tumor pathology is the gold standard for the diagnosis of recurrence, and biopsy is recommended to obtain histopathological evidence if possible (7).

In patients with localized recurrence after comprehensive treatment for rectal cancer, several studies have shown that radical resection (R0 resection) is an independent factor influencing survival in patients with localized recurrence (3, 8, 9). Direct surgical resection is recommended for patients with a history of pelvic radiotherapy for isolated tumors without distant metastases and locally resectable tumors. Based on the relevant examination results, a multidisciplinary team discussion was held to assess the R0 surgical resectability and surgical risk, determine the surgical access, choose the surgical procedure and the scope of resection, and adequately plan the surgery to minimize the occurrence of postoperative complications.

If the boundary of the lesion is unclear, the infiltration range is wide, it is difficult to resect cleanly by surgery, or the patient has absolute contraindications to radical surgery (such as severe cardiopulmonary dysfunction unable to tolerate surgery, bilateral sciatic nerves invaded by the tumor, external iliac blood vessels involved, pelvic wall invasion, etc.) (10), in those cases, surgery should not be performed immediately, and preoperative combined radiotherapy and chemotherapy should be performed so as to strive for the chance of surgery.

Due to the complexity of the pelvic anatomy and the destruction of the normal anatomical structure by the first surgery or radiotherapy, the traditional transabdominal or transabdominal perineal combined approach is difficult to perform, and the unclear surgical field, anatomical structure disorder, local adhesions, tumor infiltration, and roughness of the operation can increase the difficulty of the surgery, the intraoperative damage to the neighboring organs in the urinary tract, the risk of bleeding, and the difficulty of precise hemostasis. Therefore, improving surgical access, ensuring the curative nature of surgery as much as possible, shortening the operation time, and reducing the occurrence of complications have become the core of treatment.

The transsacral prone longitudinal incision approach (i.e., the traditional Kraske approach) (11) is commonly used in the surgical treatment of low-level presacral masses (The upper level of the mass is below S4) to achieve complete resection of the mass and to reduce local recurrences (12, 13). Many scholars have modified the Kraske approach and used it also for benign, precancerous and malignant lesions in the lower and middle rectum and for Posterior rectal tumors (14). The transsacral prone longitudinal incision approach is a well- exposed, less invasive, and inexpensive procedure. Combined with the fact that the recurrent tumor tissue of this patient is located in the posterior wall of the upper section of the vagina, the location is deep. If the patient goes through the transabdominal approach, it is more difficult to fully expose the surgical field, and it is not easy to reveal the bleeding point when bleeding during the operation, so it is difficult to completely resect the tumor tissue. If the patient goes through the transabdominal perineal approach, the surgical trauma is large, and due to the destruction of the normal anatomical structures of the pelvic cavity caused by the patient’s first surgery and radiotherapy, there is a higher risk of bleeding from the presacral venous plexus during the operation.

Therefore, after discussion by our multidisciplinary team, the patient in this case was selected for a transsacral prone longitudinal incision approach. This approach can reduce tissue damage, reduce the degree of organ function destruction, and facilitate hemostasis with a good view if intraoperative bleeding occurs. If the incision is too tense to be sutured, a bilateral gluteus maximus “kite” flap can be embedded to completely cover the trauma and promote healing of the surgical wound. For the design of flaps, gluteal perforator flap, fasciocutaneous flap and gluteus maximus muscle flap can be used for sacrococcygeal soft tissue defects with good results (15). This case was selected as a bilateral gluteus maximus kite flap combined with postoperative continuous negative pressure suction to repair soft tissue defects in the operative area. During the operation, a portion of the gluteus maximus muscle was dissected, the superior gluteal artery was preserved, the outer muscle tissue tip of the flap was extended and cut, and the flap was cross-folded and filled at the incision.

Because the gluteus maximus muscle flap is softer, it is suitable for rotational advancement and transfer; the muscle tissue at the distal end of the extended flap can fill the deep cavity well. Postoperative area can be filled with residual cavity due to abundant soft tissue filling. Combined with negative pressure drainage in the surgical area, it can form a closed negative pressure environment in the surgical area, so that the skin graft and the tissues under it are closely adhered to each other, effectively preventing the formation of dead space and the accumulation of blood and fluid under the skin graft. It can also inhibit the perfusion of CD68+ macrophages and the expression of tumor necrosis factor-α and interleukin-1β, as well as reduce the interstitial edema and apoptosis of the free flap (16). The postoperative healing of the surgical site in our patient was good, with no complications such as infection, poor incision healing, or urinary dysfunction.

Complete resection of locally recurrent tumor tissue is central to improving patients ‘ quality of life and prolonging survival. Currently, radical surgical options for local recurrence of rectal cancer are still undergoing continuous exploration to ensure radical surgical resection while minimizing complications and improving patients ‘ quality of life. For patients with local recurrence involving the vagina after comprehensive treatment of rectal cancer, if the possibility of radical resection is evaluated after discussion by a multidisciplinary team, in addition to the traditional transabdominal or combined transabdominal-perineal approach, the longitudinal incision approach through the prone position of the sacrum combined with bilateral gluteus maximus “kite” flaps is also an option, which can successfully resect the tumor in a complete manner, reduce the difficulty of the operation, postoperative healing of the operated area is well, significantly improve the quality of life and prolong the survival of patients. This surgical approach does not require special medical equipment, and clinicians need to fully understand the anatomical structure, which makes it easy to promote its application.

## Data Availability

The original contributions presented in the study are included in the article/supplementary material. Further inquiries can be directed to the corresponding author.
